# Measuring sperm backflow following female orgasm: a new method

**DOI:** 10.3402/snp.v6.31927

**Published:** 2016-10-25

**Authors:** Robert King, Maria Dempsey, Katherine A. Valentine

**Affiliations:** 1 School of Applied Psychology, University College Cork, Cork, Ireland; 2 Lecturer in Psychology at Chapman University, Orange, CA, USA

**Keywords:** Female Orgasm, Evolution, Insuck, Sperm Retention, Fertility

## Abstract

**Background:**

Human female orgasm is a vexed question in the field while there is credible evidence of cryptic female choice that has many hallmarks of orgasm in other species. Our initial goal was to produce a proof of concept for allowing females to study an aspect of infertility in a home setting, specifically by aligning the study of human infertility and increased fertility with the study of other mammalian fertility. In the latter case - the realm of oxytocin-mediated sperm retention mechanisms seems to be at work in terms of ultimate function (differential sperm retention) while the proximate function (rapid transport or cervical tenting) remains unresolved.

**Method:**

A repeated measures design using an easily taught technique in a natural setting was used. Participants were a small (n=6), non-representative sample of females. The introduction of a sperm-simulant combined with an orgasm-producing technique using a vibrator/home massager and other easily supplied materials.

**Results:**

The sperm flowback (simulated) was measured using a technique that can be used in a home setting. There was a significant difference in simulant retention between the orgasm (M=4.08, SD=0.17) and non-orgasm (M=3.30, SD=0.22) conditions; t (5)=7.02, p=0.001. Cohen's d=3.97, effect size r=0.89. This indicates a medium to small effect size.

**Conclusions:**

This method could allow females to test an aspect of sexual response that has been linked to lowered fertility in a home setting with minimal training. It needs to be replicated with a larger sample size.

Debate concerning the nature and function of human female orgasm has been intense – although frequently producing far more heat than light. According to most scholars in the field (Barash, 2005; Judson, 2005; Pound & Daly, 2000; Puts, 2006), it is still an open question whether female orgasm has been sculpted by natural selection to somehow increase the dispersal of genes in future generations or whether it exists only as a by-product of some other adaptation. In this latter case, the most promising candidate would be the strong selection on the sensitivity of the male homologue of the clitoris – namely the penis (Gould, 1987; Lloyd, 2005; Symons, 1979) whereby female orgasms exist only because male ones do – having no function of their own. A by-product account typically emphasizes the allegedly poor location of centers of female sexual sensitivity for coitally produced orgasm to occur. Proponents of this theory then typically go on to argue that female sexual organs therefore exhibit a ‘Rube Goldberg haphazardness’^1^ (Gould, 1987; Lloyd, 2005; Symons, 1979; Wallen & Lloyd, 2008).

Candidates for an adaptive function for female orgasm have broadly fallen into two types. There are those who argue that female orgasms help to cement pair bonds, in a species requiring bi-parental care (Eschler, 2004, 2005; Morris, 1967; Rancour-Laferriere, 1983). In contrast are those who assert that female orgasm allows preferential selection of sperm from any one of a multitude of partners (Baker & Bellis, 1993a, 1993b; Thornhill, Gangestad, & Comer, 1995). Sperm selection is a feature of female choice mechanisms. In contrast to Bateman's principle, there is a large body of evidence across taxa that female fitness increases as sexual partners increase, just in case that there is some mechanism for picking the best sperm among sexual partners (Simmons, 2005). Note that there is no requirement for these to be concurrent sexual partners. It need not be the case that sperm from multiple partners is simultaneously present in the female – just that there operates some sort of differential sorting mechanism that privileges some matings over others.

It could be argued that these two adaptive accounts are to some extent orthogonal to one another, generating a somewhat different set of testable predictions. For example, with respect to the pair-bond hypothesis, the finding that primate female orgasm in general seems to occur more frequently in extra-pair copulations (Ellsworth & Bailey, 2013) with high dominance or high genetic quality partners (Gangestad & Thornhill, 1995; Puts, Welling, Burriss, & Dawood, 2012; Thornhill et al., 1995; Troisi & Carosi, 1998) runs counter to prediction. However, it could also be the case that reliably produced coital orgasms tend to draw a heterosexual couple together (Rancour-Laferriere, 1983; Wheatley & Puts, 2015). Viewed in this light, the comparative difficulty in females’ achieving coital orgasm with a multiplicity of partners is a non-accidental feature of such orgasms having adaptive significance. They are part of a female choice mechanism that responds differentially to different partners. In other words – female orgasm may represent, in part, a ‘try before you buy’ mechanism that simultaneously increases conception chances and motivates the female to bond with that partner. A recent detailed and authoritative review of the literature concluded that the balance of probability was that some form of sperm-selection mechanism was the most likely candidate for the functional aspect of human female orgasm (Puts, Dawood, & Welling, 2012). However, the physiological nature of such a mechanism is still in dispute – with anatomical authorities in disagreement about whether it is the proximate mechanism of cervical tenting (Levin, 2011) or rapid transport (Zervomanolakis et al., 2007) by which this evolved function is performed.

The clitoris is crucial to understanding the nature of female orgasm. However – and contrary to much popular and scholarly opinion – the clitoris is not a Rube Goldberg contraption ill-suited to the production of orgasm through intercourse (Gould, 1987; Hite, 1976; Lloyd, 2005; Symons, 1979; Wallen & Lloyd, 2008; although see Wallen & Lloyd, 2011 for a partial retraction of this position). On the contrary – the clitoris is an exquisitely balanced measuring instrument – with at least 18 separate, but functionally, integrated components (Dickinson, 1949; Kobelt, 1851; O'Connell, Hutson, Anderson & Plenter, 1998; O'Connell, Sanjeevan, & Hutson, 2005). Most of its 4-inch length of sensitive vascular tissue is hidden inside – prompting one leading anatomist to call it an ‘iceberg organ’ (R. Levin, personal communication, July 2010). The exterior part, or glans, is highly sensitive – for example, it is a key area to be retained during genitoplasty (Schober, Meyer-Bahlburg, & Ransley, 2004) but this is by no means the only important area of female sexual sensitivity. Surprisingly, even women who have had the clitoral glans entirely, even brutally, excised still report the experience of coital orgasm (Lightfoot-Klein, 1989). In addition, it has been found that there are a variety of neural pathways in the vaginal area, independent of the clitoris, that are important in female sexual response – even to the point of triggering orgasm in cases of complete spinal transection (Komisaruk et al., 1996, 2004; Komisaruk & Sansone, 2003). Recently, these multifarious and separate neural pathways of genital sensitivity have been mapped onto the sensory cortex (Komisaruk et al., 2011), which is markedly different in males and females (Di Noto, Newman, Wall, & Einstein, 2013) arguing for separate underlying evolutionary pathways in male and female urogenitary systems.

An underlying assumption of much research into female orgasm has been that all orgasms are the same, and in this respect, the work of Masters and Johnson (1965, 1966) has been foundational. However, their pioneering research is not the last word on the topic. In particular, there are good reasons to believe that their methodology of studying unpartnered masturbation in a laboratory setting may not have quite captured all of the salient features of the human sexual experience. Attending to features of evolutionary biology would lead one to expect that sexual partner characteristics and behaviors (King & Belsky, 2012; Troisi & Carosi, 1998), as well as cognitive and emotional components of the experience (Levin, 2002; Mah & Binik, 2002), to be more than cosmetic features of sexual encounters. Specifically, humans typically perform sex in private consortships where they can focus on reproductively salient features of their prospective partners.

Although Masters and Johnson (1965, 1966) found no evidence of sperm insuck in their pioneering research, their methodology lacked key ingredients of ecological validity. First, the six experiments on which their (null) sperm insuck findings were based involved no actual intercourse at all – they involved unpartnered masturbation of the clitoral glans while a rigid transparent insertable was inside the vagina. But such insertables do not behave like penises during intercourse – which bend to the shape of the vagina (Schultz, van Andel, Sabelis, & Mooyaart, 1999) so as to be able to interact with sensitive areas of the anterior vaginal wall including the paraurethal gland (Grafenberg, 1950; Komisaruk & Sansone, 2003; Komisaruk et al., 2004; Komisaruk, Whipple, Gerdes, Harkness, & Keyes, 1997; Levin, 2002; Perry & Whipple, 1981; Zaviačič, 1999) and the full internal length of the clitoris (Dickinson, 1949; O'Connell et al., 1998, 2005). For certain key experiments, Masters and Johnson (1965, 1966) used inserted metal specula that covered all of these sensitive areas. Thus, it is likely that their methodologies effectively sidelined key features of female orgasms, and therefore, their conclusion that female orgasm was a unitary phenomenon – always the same no matter how achieved or with whom – may have reflected nothing more than an experimental artifact.

The recognition that not all female orgasms are the same is a commonplace assumption among many sex researchers (Bentler & Peeler, 1979; King & Belsky, 2012; King, Belsky, Mah, & Binik, 2011; Levin, 1981, 1998, 2001, 2004; Levin & Wagner, 1985; Mah & Binik, 2001, 2002; Singer & Singer, 1972), sex therapists (Brody, 2007; Butler, 1976; Costa & Brody, 2007; Fisher, 1973; Robertiello, 1970; Sundahl, 2003), and many – though not all – women themselves when asked directly (Hite, 1976). Female orgasms can vary in sensation, location, and important features of phenomenology. Following suggestions (Dawood, Kirk, Bailey, Andrews, & Martin, 2005; Judson, 2005), it has been found that women themselves report that their orgasms vary in type (King et al., 2011) and in ways based on how they are brought about (King & Belsky, 2012). Furthermore, this variability hints at adaptive functionality for at least one component of female orgasmic experience – that of oxytocin-mediated uterine peristalsis, a mechanism that has been hypothesized to underlie sperm insuck (King & Belsky, 2012; Wildt, Kissler, Licht, & Becker, 1998). Taken together, it is very likely that in previous studies, including those of Masters and Johnston, the full length of the clitoris, most of which is internal, had not been aroused. Some orgasms are reported as being accompanied by deep internal sensations that could be the corollary of orgasmic function itself.

Differential sperm insuck has been investigated in humans using a number of physiological measures (Fox, 1976; Fox & Fox, 1971; Fox, Wolff, & Baker, 1970; Wildt et al., 1998; Zervomanolakis et al., 2007; but see Levin (1998) for a proximately alternative, but functionally equivalent view). Using radio telemetry devices, the teams (Fox & Fox, 1971; Fox et al., 1970) found pressure changes in the uterus following coitally generated female orgasm. Although there have been some suggestions that such pressure changes could have been caused by freak weather conditions (Wallen, 2006), these would have had to have been implausibly large to have caused the measured effects and, in any case, were controlled for in the original studies and in follow-ups (Fox & Fox, 1971; Fox et al., 1970). It has been reported that the Fox et al. (1970) studies show the opposite of what they claimed (Lloyd, 2005). Specifically, some researchers have said that the Fox et al. (1970) data show pressure changes in the wrong direction (Lloyd, 2005; Wallen, 2006). However, close inspection of the original paper shows that such criticisms rest on a misreading of the original graphs – which are admittedly laid out in a somewhat confusing manner. Specifically, critics have read them as saying the opposite of what they actually showed. Usually graphs have a time line along the *x*-axis that runs with time flowing from left (time 0) to right. For one reason or another, the Fox et al. (1970, p. 246) graphs run the opposite way to custom (i.e. the *y*-axis is to the right, not the left of the graph making time run the other way). Critics who thought that the graphs showed the opposite of what the Fox et al. team showed were simply viewing them the wrong way around. Results of these studies, therefore, do suggest that pressure changes occur in the predicted direction following coitally generated orgasm. However, it must be admitted that these pioneering studies did not measure fluid movement per se – only the potential for such movement driven by uterine pressure changes.

Wildt et al. (1998) found insuck of sperm-like substances in the fallopian tube ipsilateral to the dominant follicle after administration of oxytocin. Oxytocin is a known correlate of female orgasm (Blaicher et al., 1999) and also creates the uterine peristalsis that could cause the pressure changes found by others (Carmichael, Warburton, Dixen, & Davidson, 1994; Fox & Fox, 1971). Wildt et al. (1998) describe the whole system as a peristaltic pump with clear links to fertility. For example, following administration of oxytocin, there was insuck of suitable material into the fallopian tube ipsilateral to the dominant ovary. Furthermore, pregnancy rates have been observed to be higher in those women in whom such transport could be demonstrated (Zervomanolakis et al., 2007). There has been some criticism (Levin, 2011) that the amounts of oxytocin in the bloodstream (3IU) used in such studies are unrealistically high. However, the peripheral amounts in the bloodstream are much less important than those found at the site of action – that is the uterus (L. Wildt, personal communication, August 2011).

Baker and Bellis (1993a, 1993b) assumed the insuck mechanism in their promising, and much cited, research into the putative functionality of female orgasm.^2^ However, they did not attempt any direct measures of sperm insuck – relying instead on subjective reports of sperm backflow amounts from participants and estimates of sperm quantities. Some (Lloyd, 2005) have found the Baker and Bellis (1993b) statistical analysis of these data to be confusing. For example, Lloyd (2005, p. 206) criticized Baker and Bellis (1993b) for their use of nonparametric tests and standardization of rank scores, although Baker and Bellis (1993b) describe the conditions for their analytic procedures in scrupulous detail. However, it must be admitted that their findings left the adaptive status of female orgasm theoretical rather than established (Pound & Daly, 2006) because flowback was estimated rather than directly measured. A further possible drawback to the Baker and Bellis (1993b) methodology is that they did not distinguish coital from masturbatory orgasms in terms of the likelihood of producing sperm insuck and there are reasons to distinguish female orgasm types (King & Belsky, 2012).

Sperm transport by some form of intra-uterine action is a phenomenon that has been found across mammalian taxa – as one might expect of an adaptation (West-Eberhard, 1992). It has been documented for decades in rats, cows, dogs, horses, rabbits, and macaques (Ammersbach, 1930; Evans, 1933; Genell, 1939; Goldfoot, Westerborg-vanLoon, Groeneveld, & Slob, 1980; Krehbiel & Carstens, 1939; Millar, 1952; Toner & Adler, 1986; Trapl, 1943; Van Demark & Moeller, 1951). If such transport responds differentially to salient features of the partner – in other words becomes sperm selection – then this would help complete the picture of a function for female orgasm. To clarify, it is hypothesized that at least some of the phenomenological features of female orgasm, such as feelings of deep uterine peristalsis, may be exactly as they appear to be.

In the farming industry, the generation of uterine peristalsis – via techniques that more accurately simulate insemination by a dominant male – is commonplace to increase sperm uptake through artificial insemination (Knox, 2001). If humans do not show sperm transport, then they are the exception among mammals. All of the mammals in which differential sperm transport (of which insuck could be a component) has been demonstrated show some degree of promiscuity – or, more properly, polyandrous mating. However, some have expressed skepticism that ancestral (or modern) female humans might have benefitted greatly from polyandrous behaviors (Birkhead, 2000).

For there to be a mechanism to privilege some matings over others – it follows that, over evolutionary time, enough female humans must have been engaging in enough polyandry for such an adaptation to develop. Such polyandry could well be sequential – with female orgasm acting as a ‘try before you buy’ choice mechanism or as a well to harvest sperm from preferred partners while remaining in a primary mate-ship especially under violent patriarchal conditions (Peters, 1987). Although official human polyandrous marriage systems are rare – albeit less so than is commonly thought (Starkweather & Hames, 2011) – there are a number of reasons for believing that facultative polyandry among female humans is more common than often assumed. Across taxa there are patterns of physiological adaptation that are understood in terms of responses to the degree of female polyandry. For example, monandrous gorillas have small testicles, polyandrous chimpanzees have large ones to produce the necessary amounts of material to compete effectively inside the females (Dixon, 1998). Human testicle size falls in between the extremes of monandry and polyandry (Short, 1979). There exist other similar male physiological adaptations hypothesized to be accounted for by female polyandry – such as proportionate sperm increase in multi-male, single-female, sexual encounters (Pound, 2002) and, perhaps, a penis adapted to excavate the sperm of prior partners (Gallup et al., 2003). It has also been argued that there are specific male psychological adaptations to the possibility of sperm competition, such as violent male sexual jealousy (Buss, 2000). That this jealousy is not always misattributed is evidenced by the degree of extra-pair paternity – estimated to be currently somewhere between 2 and 10% in humans (Anderson, 2006; Greiling & Buss, 2006). The lower figure is reported by said scholars as probably more accurate, but may well be high enough to drive adaptations. Consider an activity that had a 1% chance of lifetime (pre-reproduction) mortality. Zubrow (1989) showed that just such a mortality difference in two overlapping populations would extinguish one of them within 30 generations – less than a millennium.

Female responses to other females’ socio-sexual strategies, which in modern parlance has been termed ‘slut shaming’ (Magnanti, 2012), can also be taken as evidence of the need – perhaps over evolutionary time – to have developed strategic psychological responses to polyandrous female behaviors. Attacks on female reproductive reputation are effective in ways that they are not on males and are evidenced cross-culturally (Gangestad & Simpson, 1990). Some have even argued that female orgasmic response evolved in an environment of frequent multi-partner sexual encounters (Hrdy, 1986). In any case, it is generally becoming accepted that models of sexuality that imply females to be coy and passive in the face of aggressive male sexual ardor are seriously at variance with the facts – whether in humans or other animals (Gowaty, 1997; Hrdy, 1981, 1986, 1996; Judson, 2003; Zuk, 2002). Put bluntly, Bateman's principle – that females do not benefit from multiple matings – is wrong. Female choice mechanisms are key drivers in primate evolution (Cronin, 1991), and facultative polyandry is a suite of mechanisms that can instantiate such choice.

## The current study

In an effort to move the debate forward, we decided to investigate the phenomenon of sperm retention – possibly through orgasmic insuck – directly in human females. To our knowledge, this has not yet been measured directly in humans. Anywhere from 5 min to 2 h following intercourse, there is a phenomenon called backflow – an outflow of about 3 mL of material, including semen, not taken up into the reproductive tract (Baker & Bellis, 1993a).^3^ Minimizing backflow is a commonplace in the farming industry where maintenance of low backflow is highly correlated with fertility in a variety of species (Knox, 2001; Mezalira, Dallanora, Bernardi, Wentz, & Bortolozzo, 2005; Steverink, Soede, Bouwman, & Kemp, 1998). In humans, backflow is detectable by the female in question (Baker & Bellis, 1993b) but is not under conscious control. Measurement of backflow provides a way to compare the amount of sperm taken up following orgasm, even of different kinds of female orgasm, perhaps produced in different ways.

## Method

### Participants

This exploratory study involved six females, mean age 39.3 years (SD 10.2), median age 39.5 years, age range from 26 to 52. All were white, European, or American and had been educated to at least undergraduate degree level. None were using oral contraceptives. All were sexually active with a current (male) partner. Five of the six reported that they experienced regular periods. They all reported as being either 0 or 1 on the standard Kinsey scale for sexuality. All had previously reported variation in orgasmic phenomenology (e.g. deep or shallow orgasms) in line with previous findings (37). They were recruited via snowballing of interested parties, accessed via personal connections or interests. There was one dropout during the testing period (e.g. from an original seven). All participants who remained reported that the procedure was ‘easy’ or ‘fairly easy’ to carry out. While the ‘masturbation not leading to orgasm’ condition (detailed below) did create some issues of sexual frustration, all participants retained in the study reported success with the procedure. For further details of questions asked, see Appendix 1.

A sample of the instructions for performing the procedure, including an instructional video, is available from the authors on request. The study was carried out under the supervision of the Ethics Board of University College Cork (approved 15/03/2013). Participants were fully debriefed, and arrangements were made for them to be referred to specialist support if issues of inorgasmia were raised (which did not occur).

### Materials

#### Properties of human semen

Human semen has a number of chemical and physical properties. Of the former, pH and buffering capacity, typical citrate concentration, ion composition (calcium, magnesium, potassium, sodium and zinc), osmolarity, sugar components (fructose and glucose), and, finally, protein (albumin) concentration have all been assayed in considerable detail (Owen & Katz, 2005). Important physical properties of human semen include typical volume and viscosity. Typical ejaculate volume has been measured to have a range between 2.7 mL (Bhushan, Pandey, Singh, Pandey, & Seth, 1978) and 4.99 mL (Purvis, Magnus, Morkas, Abyholm, & Rui, 1986). The higher figures are typically, though not universally, found for semen collected during copulation rather than following masturbation (Owen & Katz, 2005).

Measurement of viscosity is harder to specify as the rheological properties of semen change after ejaculation. The initial ejaculate coagulates and then subsequently liquefies in from anywhere between 5 min (*in vivo*) and 30 min (*in vitro*) (Montagnon, Clavert, & Cranz, 1982; Polak & Daunter, 1989). Semen has a number of (often non-Newtonian^4^) viscosity properties such as its elasticity, thixotrophy, shear thinning, and yield stresses, which are not captured by the standard World Health Organization (WHO) (1999) laboratory standard measures of viscosity (see Owen & Katz, 2005, for a more extended discussion). In their authoritative exposition of the production of an effective semen stimulant, authorities in the field (Owen & Katz, 2005) settle on a viscosity of 1.3 cP.^5^

#### Semen simulants

There are a number of products on the market that reasonably closely approximate to the viscosity of human semen. Water-based lubricants are preferred for a number of reasons including hygiene and health. Lubricants intended for use in sex closely – and non-accidentally – approximate to the human mucin viscosity levels. In particular, the Bodywise™ company produce a water-based lubricant, called Liquid Silk™, which is also dermatologically neutral and safe for ingestion.

#### Collection devices

The Mooncup^(TM)^, manufactured by Bodykind ™, is a device used as an alternative to tampons and sanitary pads for collecting menstrual flow in an environmentally friendly and hygienic fashion. It is a medical-grade silicone cup – about the size of an eggcup – that can be held in place during a woman's period. Being unobtrusive and comfortable to wear, it is also ideally suited to collecting backflow over the up to 2 h time period necessary to do so.

In addition, each woman was supplied with a sterile 10 mL syringe body, which was to be used for both introduction and collection of the sperm stimulant, surgical glove, and spoon.

### Procedures

The methodology was explained to each female in the same way so as to standardize procedures as far as possible. There was a trade-off between the requirements of ecological validity – that orgasm be as natural as possible – and the demands of scientific standardization of procedures. Actual coition introduces a range of unacceptably uncontrollable variables – a particularly crucial one being ejaculate quantity. However, previous research had found that normally achieved masturbatory orgasms were insufficient to produce the uterine peristalsis that is hypothesized to generate insuck (Masters & Johnson, 1965).

Fortunately, technology has moved on since the days of Masters and Johnson (1965). In particular, devices such as the Hitachi Magic Wand™ can produce deep tissue stimulation from the outside due to the tremendous amount of (mains) electricity power used – producing somewhere in the region of 6,000 RPM. Previous interview-based research (King & Belsky, 2012) has revealed that deep peristaltic effects during orgasm were common – although not universal – with such devices. Here, the pornographic film industry is ahead of science. The easiest way to create the convincing orgasms in female actors (Saad, 2011) is to have actual orgasms occur. To some customers, this is a crucial issue and a common topic of discussion on Internet message boards. The Hitachi Magic Wand™ is one of the most common devices for creating such sensations and effects. This device allowed us to produce orgasms with the phenomenology of deep internal uterine peristalsis while controlling for amount of sperm simulant introduced – something that regular coition would not allow.

A potentially important control was estral timing. If orgasm is connected to fertility, then it might be predicted that it occurs differently at peak fertility. Thus, each female collected backflow on three separate occasions – at the end of the flow period, at peak estrus, and, finally, just before the start of the next period. It was left up to the women themselves to decide this. They kept diaries of their periods and all reported regularity with them. Emphasis was on collection at peak estrus, given that theory predicts that this is when any differential sperm selection would occur – if it does occur (Baker & Bellis, 1993b). In addition, females filled in a questionnaire that asked for phenomenological details of the orgasm in ways that have been previously found to be of significance (King & Belsky, 2012). This is because it has been proposed that the putative insuck component of female sexual response does not occur at every orgasm (King & Belsky, 2012; King et al., 2011).

#### Conditions

The most important comparisons were between orgasm achieved through masturbation of clitoral glans and masturbation that did not lead to female orgasm. This is thus a repeated measures design – with each participant acting as her own control.

Prior to each condition, the female herself introduced 5 mL of appropriate measuring substance (see above) and then either masturbated to orgasm, or not to orgasm, as a control – in as far as possible for the same amount of time. The use of a suitable deep-tissue massager as detailed above was used. As soon as activity had finished, the woman inserted the Mooncup^(TM)^ and waited a suitable time established in pre-testing for backflow to occur – 1 h. The resultant substance was then extracted and measured immediately (using the same measuring syringe) to avoid loss of fluid due to evaporation. Given the nature of the collection, it was not possible that the participants be blind to the research hypothesis. However, by standardizing procedure as far as possible, it was hoped that any biases be minimized as far as realistically possible. After a brief period of pre-test consultation and feedback from participants, the procedure was altered to emphasize that orgasms that produced female ejaculate should not be included in the results.

#### Protocols

This was a repeated measures design with each participant acting as their own control. The independent variable was simple – orgasm (or not) immediately following introduction of sperm-like material. In order to control for participant variables (such as desire), the decision as to whether an orgasm would occur was decided by a single coin flip 1 h prior to each collection exercise. It was left to participants to find convenient times for collection to occur, within the parameters of estral timing. The procedure continued until there had been half of the possible collections under one set of conditions, then the rest were carried out. There was thus a (more or less) randomized element introduced to try to control for extraneous variables. The control was the no orgasm condition – which still involved introduction of material and subsequent measuring. The measurements taken here provided the baseline for comparison. Introduction of sperm simulant was achieved through the use of a sterile syringe body (5 mL). Following said introduction, the collecting device described above was applied and an hour was allowed to pass. Instructions were that once a quota of orgasm or non-orgasm conditions had been achieved by random coin flip, then the remainder of the protocols would be of the missing condition.

During this collection time, the participant went about normal business (which the Mooncup™ allows for). Although some (Morris, 1967) have averred that female orgasm creates drowsiness – the so-called poleax effect – this has not been the finding of any other scholars in the field (in other words no one else of whom we are aware has reported the hypothesized drowsiness) and any putative sperm retention mechanisms should work irrespective of gravity. Strenuous post-coital activity was avoided, however. After an hour, the Mooncup™ was removed and the residue inside was measured by using (now cleaned) syringe body to draw in any resultant material and the quantity read off from the side. Five measurements of each condition were taken, including three during the peak estrus period – as estimated by counting forward to mid-cycle. This is held to be an acceptable number of measures to be taken to reduce random errors (Kirkup, 1994). Measurements taken at the start and end of flow period were averaged as there were no visible differences between the amounts measured. No measurements were taken at the time of each woman's period. All participants were non-users of female oral (i.e. hormonal) contraceptives. Findings were recorded in the manner detailed below (Appendix 2). Each participant also recorded phenomenological details of the orgasms produced.

## Results

Sperm backflow was measured using the protocol detailed above. Given that a measured dose of 5 mL was introduced in each case, higher numbers indicate larger quantities of the sperm simulant being retained. Table 1 shows the comparison between the (presumed) high and low fertility conditions in terms of retention of sperm simulant.

**Table 1:** Comparison of high/low fertility conditions following orgasm in terms of simulant retention

	Orgasm (high fert)	No orgasm (high fert)	Orgasm (low fert)	No orgasm (low fert)
Participant				
1	4.0	3.5	4.1	3.6
2	4.2	3.0	4.2	3.0
3	4.2	3.6	4.2	3.2
4	4.1	3.5	4.2	3.5
5	3.8	3.2	3.7	3.2
6	4.2	3.0	4.1	3.3
M (SD)	4.1 (0.16)	3.3 (0.27)	4.1 (0.19)	3.3 (0.22)

If there were any differences between high and low fertility conditions, these were undetectable given the methods used. The initial intent was to be able to look at results by hypothesized high/low fertility group. However, given the small *N*, the low fertility/high fertility figures were merged to create an average sperm retention score allowing for more repeated measures data points to maximize power. It may well be that follow-up research finds a significant difference here. However, it was drawn to our attention that the window for human female fertility may well be broader that is commonly thought and that sperm stays viable in the reproductive tract for a surprisingly long time (Gould, Overstreet & Hanson, 1984). Given all this, plus the exploratory nature of the study, it was considered reasonable to analyze the results as a unit despite the fact that the initial intent had included a test of the putative difference between high/low fertility conditions. It seemed dishonest to pretend that our initial intent was otherwise. Table 2 represents the difference between the orgasm and non-orgasm conditions expressed as a percentage of the total sperm-simulant insertion (5 mL in each case). Results of the questionnaire (closed questions) revealed that all the orgasms in question were experienced as deep inside.

**Table 2:** Comparison of retention of sperm simulant in orgasm/no orgasm conditions

	Orgasm (mL)	No orgasm (mL)	Percentage difference retained
Participant			
1	4.05	3.55	10
2	4.20	3.00	24
3	4.20	3.40	16
4	4.15	3.50	13
5	3.75	3.20	11
6	4.15	3.15	20
M (SD)	4.1 (0.17)	3.3 (0.22)	15.7

Although sample sizes were not large enough for rigorous frequentist hypothesis testing (Tabachnick & Fidel, 2007), there are still things about the data that can be explored statistically. The differences between the hypothesized high and low fertility conditions were not significant but comparisons were made between the aggregated orgasm and non-orgasm conditions for each of the six participants. Treating the combined orgasm and non-orgasm conditions as paired samples showed that there was a significant difference in simulant retention – measured in terms of lower amount of flowback – between the orgasm (*M*=4.08, SD=0.17) and non-orgasm (*M*=3.30, SD=0.22) conditions: *t*(5)=7.02, *p*=0.001. Cohen's *d*=3.97, effect size *r*=0.89. This indicates a medium to small effect size.

## Discussion

The goal of the research was to establish a workable method for testing the existence of and measuring the result of sperm-retention mechanisms in human female orgasm – bringing it in line with fertility-related research in other species. Our aim was to generate a protocol that could be carried out easily and at home and that might be of use and interest to a wider audience than those with the specific interest of understanding human female sexual response – that is those with a practical interest in their own fertility.

The protocols outlined here suggested a mechanism that can be used to assess differing hypotheses in respect of human female orgasm. Furthermore, it appears that female orgasm does perform some sort of sperm-retention function. These results were in line with what has been found in respect of sperm retention following oxytocin-stimulating procedures in other species (Knox, 2001). However, there are some important caveats.

One major limitation is that the sample size is, at present, very small. While this is in line with the sample sizes in other studies in human female orgasm otherwise taken to be authoritative (e.g. Masters & Johnson (1965) used six participants in their key study), the procedure presented here should be taken as a proof of concept and of procedural validity, rather than as conclusive evidence of a sperm-retention function for human female orgasm. The sample size was insufficient to produce anything more than (albeit highly suggestive) descriptive statistics. While the average effect size of putative sperm-retention was large (15.7%) and in line with that found in other species (Knox, 2001), these are preliminary findings only. There is a need for these findings to be replicated in a larger sample.

Furthermore, volunteers for a study such as this are highly unlikely to be representative of the female population at large. They are likely to be more generally sex-positive (Plaud, Gaither, Hegstad, Rowan, & Devitt, 1999; Strassberg & Lowe, 1995) and more generally novelty-seeking (Wiederman, 1999). However, even admitting this, it is unlikely that any of these things would have much effect on the mechanisms under investigation. However, the lack of blinding of the procedure may have led to biased measurement – especially as some volunteers were recruited via personal connections and interests, while others approached one of the authors following a discussion and request for volunteers on a blog post about sexual research. Such volunteers may be motivated, albeit subconsciously, to bias in their measurements. We hope that the procedure is sufficiently robust and simple enough to be carried out by a larger sample of parties interested in the fertility-related aspects of their sexual response and look forward to independent replication. This would help to offset the necessarily invasive elements of the study protocol.

In terms of future directions, even if human female orgasm is established (through subsequent study) to be sperm retaining, the precise mechanism of retention is still disputed by physiological scholars in the field. Some have argued that during female orgasm, the cervix tents slowing sperm intake and thereby increasing fertility (Levin, 1998). Others have argued that female orgasm, or at least the oxytocin release associated with it, facilitates rapid transfer of sperm (Wildt et al., 1998; Zervomanolakis et al., 2007). Either of these sperm-retaining proximate mechanisms would serve the same ultimate function – of increasing fertility – if orgasms occur selectively. And this, they appear to do (Gangestad & Thornhill, 1995; King & Belsky, 2012). The evolutionary function (if any) and the proximate mechanism (whichever it may or may not be) need to be kept conceptually distinct in people's minds lest huge confusions occur.

One finding worthy of note is that some support for taking self-report more seriously than some scholars might assume. Data and theory should be mutually supporting and build on one another. The small sample of women did report (from the relevant closed question in the questionnaire, Appendix 2) that they experienced their orgasms deep inside – what is sometimes, albeit erroneously, called a ‘vaginal orgasm’. As argued above, all orgasms are clitoral but many do not engage the full extent of the clitoris. This tends to support the idea that female orgasms that involve arousal and stimulation of the whole of the clitoris, rather than just the glans, may be important. In this respect, it could be noted that it is possible to generate ejaculation and orgasms from a flaccid male penis by using sufficient direct stimulation. It might thus be argued that any typology of orgasm is redundant. There is some merit to this argument. In support of the typology approach, it should be mentioned that it is women themselves that report this difference (King & Belsky, 2012).

Finally, sperm retention leading to increased fertility is not just of abstract or academic interest. Female orgasm is closely linked to couple satisfaction. In addition, if sperm retention does occur, then a number of interventions relating to persistently infertile couples suggest themselves. Indeed, a prominent fertility specialist is on record as saying that it is his belief that female inorgasmia explains some of the variance in fertility once other variables have been controlled for (Winston, 2010). The procedures outlined here could easily be envisaged to form part of the protocols to assist fertility in some couples as well as providing a procedure for assessing the function of female orgasm. As such, we submit that there is a proof of concept of sperm retention function of female orgasm, and a method for investigating the function of the same could be developed.

## Footnotes

1 Rube Goldberg was a cartoonist who drew fantastical machines that humorously completed mundane tasks with unnecessary complexity. The English equivalent is Heath Robinson.

2 Although they changed the terminology from ‘insuck’ to ‘upsuck’.

3 Although, for some reason, they use the term ‘flowback’ instead of the more common ‘backflow’.

4 Newtonian viscosity is inversely proportional to shear stress and directly relative to the force applied.

5 This can then be increased to 4 cP if necessary, for some coagulation studies, by the addition of methyl cellulose.

## Appendix 1

**Female Sexual Response Questionnaire**

*The information gathered through this study will be reported anonymously. Please answer the following questions honestly. While we would like you to answer all questions, if any of them make you feel uncomfortable please move to the next question*.

Demographic details

 1) Age (years) _____________

 2) Nationality _____________

 3) Number of children (if any) _________________

    Age(s) of children _________________

 4) Do you still menstruate? *Circle as appropriate* Y/N

 5) How would you describe your own sexual preferences:

   0 Exclusively heterosexual [ ]

   1 Predominantly heterosexual, only incidentally homosexual [ ]

   2 Predominantly heterosexual, but more than incidentally homosexual [ ]

   3 Equally heterosexual and homosexual [ ]

   4 Predominantly homosexual, but more than incidentally heterosexual [ ]

   5 Predominantly homosexual, only incidentally heterosexual [ ]

   6 Exclusively homosexual [ ]

6) Number of siblings (if any) _________________

 7) What is the approximate population of the place where you live? Please tick one box

   a) Less than 1,000          [ ]

   b) More than 1,000 but less than 10,000 [ ]

   c) More than 10,000         [ ]

 8) What is the highest level of education you have attained?

 ________________________________________________________________________________________________

**Pre-info Session Questions**

 9) When you first heard about the study what was your initial thoughts? *Be as frank as possible*

________________________________________________________________________________________________

________________________________________________________________________________________________

________________________________________________________________________________________________

 10) In reflecting on your sexual history has there been a time when you have you experienced recurring problems achieving orgasm? Y/N

 11) Currently, do you experience ease in achieving orgasm? Y/N

 12) How do you feel about being here tonight at the study information evening?

________________________________________________________________________________________________

________________________________________________________________________________________________

________________________________________________________________________________________________

13) How do you feel when you think about research into human sexual behavior? *Please circle as many as is relevant*

Unsettling   Exciting   Invasive   Adventurous   Salacious

Voyeuristic   Titillating   Needed   Messy   Liberating

Voyeuristic   Titillating   Needed   Messy   Liberating

Are there any other words that capture your feelings more accurately? ________________________________________________________________________________________________

**
Post-info Session Questions**

*Remember while we would like you to answer all questions, if any of them makes you feel uncomfortable, in any way, please do not feel that you have to answer them*

 14) Now that you have more information on the study, have your thoughts about it changed in any way? Y/N If yes, how have they changed?

________________________________________________________________________________________________

________________________________________________________________________________________________

________________________________________________________________________________________________

________________________________________________________________________________________________

________________________________________________________________________________________________

15) Following the information presented this evening, how do you feel now about research into human sexual behavior?

________________________________________________________________________________________________

________________________________________________________________________________________________

________________________________________________________________________________________________

________________________________________________________________________________________________

________________________________________________________________________________________________

16) Has any of the information presented this evening changed how you feel about your own sexual activity? Y/N
   

 If yes would you like expand on this a little?

________________________________________________________________________________________________

________________________________________________________________________________________________

________________________________________________________________________________________________

________________________________________________________________________________________________

________________________________________________________________________________________________

**Many thanks for being here this evening and for responding to this questionnaire**

## Appendix 2

**Table:** Sample recording table

Expt #	Date	End date of last period	PP#	Peak estrus (Y/N)	Qty inserted	After 1 h	Orgasm? (Y/N)	Deep? (Y/N?)
1	DD/MM/YY	DD/MM/YY	X	Y or N	5 ml	(<5 ml)	Y/N	Y/N
2								
3								
